# Primary Cilia Are Required for Efficient BMP Signaling in Traumatic Heterotopic Ossification

**DOI:** 10.3390/biomedicines14030712

**Published:** 2026-03-19

**Authors:** Xinyuan Yuan, Saman Toutounchi, Susan F. Law, David Achudhan, Abhishek Chandra, Kai He, Yingshu Cao, Jinghua Hu, Robert J. Pignolo, Haitao Wang

**Affiliations:** 1 Department of Physiology and Biomedical Engineering, Mayo Clinic, Rochester, MN 55905, USA; 2 Department of Medicine, Mayo Clinic, Rochester, MN 55905, USA; 3 Department of Biochemistry and Molecular Biology, Mayo Clinic, Rochester, MN 55905, USA

**Keywords:** primary cilia, traumatic heterotopic ossification, tenocytes, bone morphogenetic protein (BMP) signaling

## Abstract

**Background/Objectives:** Heterotopic ossification (HO), the aberrant formation of bone within soft tissues, arises either from rare genetic mutations or more commonly from traumatic insults. It is a major cause of morbidity not only in individuals harboring causative mutations, but also in those undergoing musculoskeletal surgery or trauma and in soldiers sustaining blast or burn injuries. Bone morphogenetic protein (BMP) signaling is a central driver of both hereditary and acquired forms of HO. Primary cilia are nonmotile, antenna-like organelles that extend from the cell surface and serve as crucial sensory and signaling hubs by concentrating key pathway components within a confined volume at the ciliary tip. However, their functional role in the pathogenesis of traumatic HO remains poorly understood. **Methods:** We investigate the role of primary cilia in traumatic HO using a genetically modified mouse model and cellular model. **Results:** We demonstrate that BMP signaling is attenuated when primary cilia function is disrupted. Both ciliation frequency and ciliary length were reduced in *Scleraxis*-CreERT2*; Intraflagellar transport 88* ^floxed/floxed^ (*Scx*-CreERT2;*Ift88*^fl/fl^) tenocytes. Deletion of *Ift88* effectively suppressed pathological BMP signaling and inhibited HO formation. **Conclusions:** These findings establish that functional primary cilia are required for traumatic HO development and highlight ciliary regulation as a potential therapeutic avenue for preventing or mitigating post-traumatic HO.

## 1. Introduction

Heterotopic ossification (HO) is a pathological condition characterized by the formation of mature bone within soft tissues outside the normal skeletal framework. It can arise from rare genetic mutations or be triggered by more common forms of tissue injury and trauma [1]. HO poses a substantial risk of functional impairment, affecting individuals with inherited predisposition as well as patients undergoing invasive musculoskeletal procedures. In addition, HO is highly prevalent among military personnel exposed to modern combat injuries, particularly those involving high-energy blast trauma and extensive burns.

Based on etiology, HO is broadly divided into hereditary and nonhereditary forms. Hereditary HO is exemplified by fibrodysplasia ossificans progressiva (FOP), an extremely rare congenital disorder caused by a gain-of-function point mutation (R206H) in the Activin receptor type I gene (*Acvr1*, also known as *ALK2*) [2]. Clinically, FOP is marked by episodic heterotopic bone formation in skeletal muscles, tendons, and other connective tissues, ultimately leading to progressive and irreversible loss of mobility [1,3]. Mechanistically, FOP mutations result in constitutive activation of ACVR1 and enhanced sensitivity to Bone Morphogenetic Protein (BMP) signaling [4,5,6,7,8,9]. Although Activin A (Act A), a member of the transforming growth factor-beta (TGF-β)/BMP superfamily, normally binds ACVR1 to inhibit BMP signaling [10,11,12], pathogenic *ACVR1* mutations in FOP convert this inhibitory signal into aberrant activation of downstream BMP effectors SMAD 1/5/8 [10,13]. This dysregulated BMP signaling drives inappropriate chondro-osseous differentiation of progenitor cells during tissue repair, culminating in the replacement of soft tissues with structurally normal bone [14,15].

In contrast, nonhereditary or acquired HO is far more common and typically develops in muscles, tendons, or ligaments following tissue injury [16]. Acquired HO frequently occurs in association with orthopedic procedures—most notably hip arthroplasty, which accounts for approximately 40% of cases [17,18,19]—as well as fractures or dislocations (~30%) [20], elbow trauma [21], high-energy extremity trauma [22], traumatic brain or spinal cord injury (with an incidence of up to 50% in spinal cord injury patients) [23], and severe burns (∼20% of third-degree burns) [24]. Among patients with severe combat-related traumatic amputations, the incidence of HO exceeds 90% [25]. Surgical excision remains the primary treatment for established lesions; however, it is associated with substantial morbidity, and the biological mechanisms underlying acquired HO remain incompletely understood [26]. Consequently, effective therapeutic options for non-genetic HO are limited.

BMP signaling has emerged as a central pathway in both hereditary and acquired forms of HO. In FOP, the canonical R206H mutation and all reported *ACVR1* variants lead to loss of autoinhibitory control and persistent activation of BMP signaling [2,27]. Similarly, in trauma-induced HO, the injury-associated stem cell niche has been shown to be BMP-dependent [28], suggesting a partially shared mechanistic basis across HO subtypes.

Primary cilia are solitary, non-motile, microtubule-based organelles that extend from the surfaces of most mammalian cells and function as critical sensory and signaling centers. By concentrating signaling components within a confined subcellular domain, primary cilia enable cells to respond efficiently to low levels of extracellular ligands [29,30]. They play essential roles in regulating key developmental and homeostatic pathways, including Hedgehog, Wnt, and TGF-β signaling [31,32]. *Intraflagellar transport protein 88 (Ift88)* is a core component of the intraflagellar transport machinery and is indispensable for ciliogenesis across species [33]. Conditional *Ift88* knockout mouse models have therefore provided a powerful approach for interrogating cilia-dependent signaling mechanisms in disease contexts [34]. In addition to BMP signaling, other cilia-dependent pathways, including Hedgehog and Wnt signaling, have been implicated in injury-induced HO and in genetic disorders such as progressive osseous heteroplasia [35]. We previously demonstrated that primary cilia regulate skeletogenic BMP and Hedgehog signaling in FOP-associated HO [36]; however, the contribution of primary cilia to BMP signaling in tenocytes during traumatic HO remains poorly defined. Elucidating the spatial and temporal regulation of BMP signaling in injury-responsive tenocytes is therefore critical for understanding the pathogenesis of HO following trauma.

## 2. Materials and Methods

### 2.1. Animal Models

All animal procedures were approved by the Mayo Clinic Institutional Animal Care and Use Committee. Mice were maintained in a temperature-controlled environment (23–25 °C) under a 12 h light/dark cycle and were provided standard laboratory chow (PicoLab Rodent Diet 20 #5053; LabDiet, St. Louis, MO, USA) and water ad libitum. *Scx*-CreERT2 mice [B6(129S4)-*Scx*^tm2.1(cre/ERT2)Stzr^/J, Jackson Laboratory] express a tamoxifen-inducible Cre recombinase (CreERT2) under the control of the endogenous Scleraxis (*Scx*) promoter, which drives expression predominantly in tendon and tenocyte lineages [37,38]. *Ift88*^fl/fl^ mice carry loxP sites flanking essential exons of the *Ift88* gene, enabling conditional deletion upon Cre-mediated recombination. *Scx*-CreERT2;*Ift88*^fl/fl^ mice were obtained by crossing *Scx*-CreERT2 transgenic mice with *Ift88* floxed mice. Thus, *Scx*-CreERT2;*Ift88*^fl/fl^ mice were used as a genetic background to obtain tendon-lineage cells, while Ift88 deletion was induced in vitro to allow for controlled analysis of cilia loss in primary tenocytes.

### 2.2. Mouse Burn–Tenotomy-Induced HO

Seven–nine-week-old male WT and *Scx*-CreERT2;*Ift88*^fl/fl^ mice (~20 g) were used for all experiments. Animals were anesthetized with 2.5% inhaled isoflurane, and the left hindlimb (knee to ankle), as well as a 2 cm × 3 cm region along the left paraspinal area, were shaved and disinfected. A longitudinal incision was made over the Achilles tendon to expose the posterior tibial tendon, which was transected transversely. The incision was closed using one or two interrupted 5-0 sutures. To induce burn injury, a 60 °C aluminum block (2 cm × 3 cm) was applied to the left side of the spinal midline for approximately 17 s without added pressure. Eight weeks after surgery, the entire left hindlimb was harvested and analyzed by micro-computed tomography (μCT) to assess heterotopic bone formation.

### 2.3. Isolation of Primary Tail Tenocytes

Primary tenocytes were isolated from the tails of one-month-old mice using a two-step enzymatic digestion protocol. Tissues were first incubated with 0.25% trypsin for 30 min to remove surface-associated cells. The remaining tendon tissue was then digested with collagenase type I and dispase II (0.2% and 0.3%, respectively) for 2 h to degrade the extracellular matrix and release resident tenocytes. Isolated cells were cultured in high-glucose DMEM (Gibco, Grand Island, NY, USA) supplemented with 10% fetal bovine serum and 1% penicillin–streptomycin. Because donor mice were not treated with tamoxifen prior to tenocyte isolation, Cre-mediated recombination was induced in vitro using adenoviral Cre; this approach was employed solely to delete *Ift88*, and potential nonspecific effects of viral exposure cannot be entirely excluded.

### 2.4. Western Blotting

Cultured cells were washed with cold PBS and lysed in 1.5× RIPA buffer supplemented with protease and phosphatase inhibitors. Protein concentrations were quantified, and equal amounts of lysates were resolved by SDS–PAGE (Thermo Fisher Scientific, Waltham, MA, USA, 1862495) and transferred onto PVDF membranes. Membranes were probed with antibodies against β-actin, p-SMAD 1/5 (Cell Signaling Technology, Danvers, MA, USA, 3700) for β-actin, total SMAD 1 (Cell Signaling Technology, 6944), and IFT88 (Proteintech, Rosemont, IL, USA, 13967-1-AP), followed by appropriate HRP-conjugated secondary antibodies. Protein signals were detected using enhanced chemiluminescence and visualized using a ChemiDoc™ MP Imaging System (Bio-Rad Laboratories, Hercules, CA, USA).

### 2.5. RT-qPCR

Total RNA was extracted from cultured cells using TRIzol reagent according to the manufacturer’s instructions. RNA concentration and purity were assessed using a NanoDrop spectrophotometer (Thermo Fisher Scientific, Waltham, MA, USA). First-strand cDNA was synthesized from 500 ng of total RNA using a high-capacity reverse transcription kit. Quantitative real-time PCR was performed using SYBR Green chemistry on a QuantStudio™ 7 Pro Real-Time PCR System (Thermo Fisher Scientific, Waltham, MA, USA). Primer sequences are listed in Table 1. All reactions were performed in triplicate, and gene expression levels were normalized to GAPDH. Relative expression was calculated using the 2^−ΔΔCt^ method, and data were analyzed using GraphPad Prism 10.

### 2.6. Immunofluorescence

Primary tail tenocytes isolated from *Scx*-CreERT2;*Ift88*^fl/fl^ mice were transduced overnight with Ad5-CMV-Cre-eGFP (Baylor College of Medicine) at a multiplicity of infection (MOI) of 1000 when cells reached approximately 80% confluence. Transduction efficiency was assessed using a fluorescence microscope (EVOS M5000 Imaging System; Invitrogen, Thermo Fisher Scientific, Carlsbad, CA, USA). Following transduction, *Ift88*-deficient cells were serum starved for 48 h and subsequently treated with recombinant BMP4 (50 ng/mL; R&D Systems, Minneapolis, MN, USA) for 1 h. Cells were then fixed in cold methanol for 30 min at −20 °C and permeabilized with 0.1% Triton X-100 for 10 min at room temperature. After blocking with 3% bovine serum albumin (BSA) for 2 h, cells were incubated with primary antibodies at 4 °C overnight, followed by incubation with fluorophore-conjugated secondary antibodies (Goat anti-rabbit IgG Alexa Fluor 647 (Cat. No. A21246, Invitrogen, Carlsbad, CA, USA) and goat anti-mouse IgG Alexa Fluor 488 (Cat. No. A10667, Invitrogen, Carlsbad, CA, USA) for 2 h at room temperature. Primary antibodies were used at the following dilutions: acetylated α-tubulin (1:500; Cat. T7451, Sigma-Aldrich, St. Louis, MO, USA) and phosphorylated SMAD 1/5 (1:500; Cat. 6944, Cell Signaling Technology, Danvers, MA, USA). Fluorescence images were acquired using a Nikon ECLIPSE Ti microscope, and quantitative analysis was performed using NIS-Elements software 5.21.03 (Nikon Corporation, Tokyo, Japan). Only clearly identifiable, intact primary cilia labeled by acetylated α-Tubulin were included in the analysis. For each experimental condition, ciliary length was quantified from 100 cilia obtained from 3 independent experiments, and measurements were performed in a blinded manner when applicable.

### 2.7. Immunohistochemistry

Formalin-fixed, paraffin-embedded tissue specimens were sectioned at a thickness of 5 µm. Hematoxylin and eosin (H&E) staining, Alcian Blue staining, and p-SMAD 1/5 staining were performed as previously described [39].

### 2.8. Micro-Computed Tomography

Hindlimbs harvested from WT or *Scx*-CreERT2;*Ift88*^fl/fl^ mice following burn–tenotomy were analyzed by micro–computed tomography (µCT) using a VivaCT 40 micro-CT system (Scanco Medical AG, Brüttisellen, Switzerland). Heterotopic bone volume and two-dimensional medial sagittal plane images were obtained for each limb. Scanning parameters included a source voltage of 55 kV, a current of 142 μA, and an isotropic voxel size of 10.5 μm. Mineralized tissue was distinguished from non-mineralized tissue using a lower threshold of 200 and an upper threshold of 1000 Hounsfield units.

### 2.9. Statistical Analysis

All statistical analyses were performed using GraphPad Prism 10 (GraphPad Software, Boston, MA, USA). Comparisons between two groups were conducted using a two-tailed unpaired Student’s *t*-test. A *p* value < 0.05 was considered statistically significant. Significance levels are indicated as follows: *p* < 0.05; * *p* < 0.01; ** *p* < 0.001; ns, not significant. Data are presented as mean ± SEM, and sample sizes are provided in the corresponding figure legends.

## 3. Results

### 3.1. Isolation and Confirmation of Primary Tenocytes

**Figure 1:**
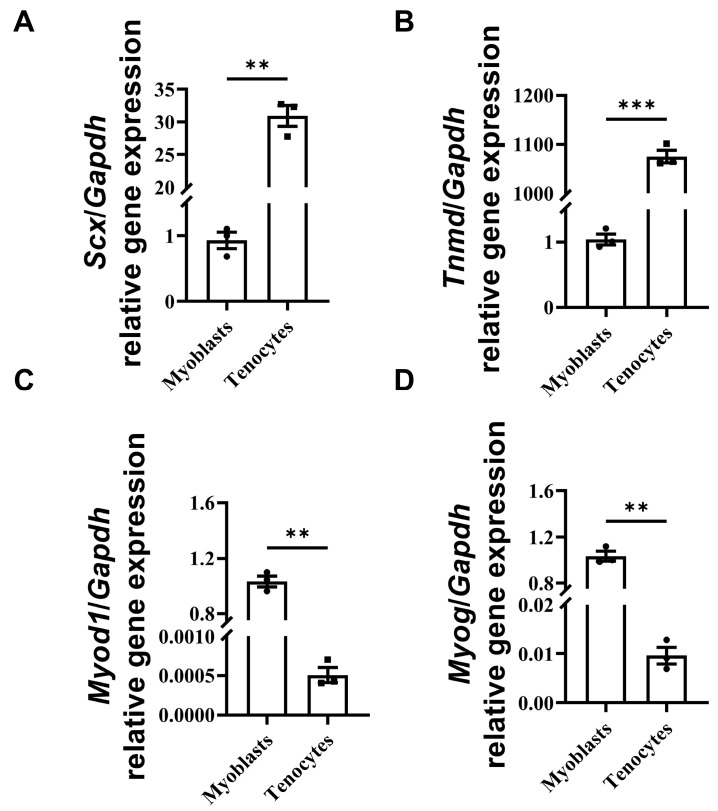
Isolation and confirmation of primary tenocytes. Primary tenocytes were isolated from tails of WT or *Scx*-CreERT2;*Ift88*^fl/fl^ mice. Expression of tenocyte and myoblast marker genes were examined. (**A**,**B**) *Scx* and *Tnmd* genes are highly expressed in tenocytes, but not in myoblasts. (**C**,**D**) the *Myod1* and *Myog* genes are highly expressed in myoblasts, but not in tenocytes. Data are presented as means ± SEM (*n* = 3). Statistical analyses were performed by two-tailed unpaired Student’s *t*-test. ** *p* < 0.01; *** *p* < 0.001.

We isolated primary tenocytes from tails of WT and *Scx*-CreERT2;*Ift88*^fl/fl^ mice. After isolating primary tenocytes, we examined the tenocyte gene expression using myoblasts as a control. The isolation of myoblasts was performed as previously published [40]. We found that *Scx* and *Tnmd* genes are highly expressed in tenocytes, but not in myoblasts (Figure 1A,B). In contrast, the *Myod1* and *Myog* genes are highly expressed in myoblasts, but not in tenocytes (Figure 1C,D). We cannot exclude the presence of minor non-tenocyte populations; however, these would be expected to be limited and unlikely to account for the observed phenotypes.

### 3.2. Ciliation Frequency and Cilia Length Decreased in Scx-CreERT2;Ift88^fl/fl^ Tenocytes

**Figure 2:**
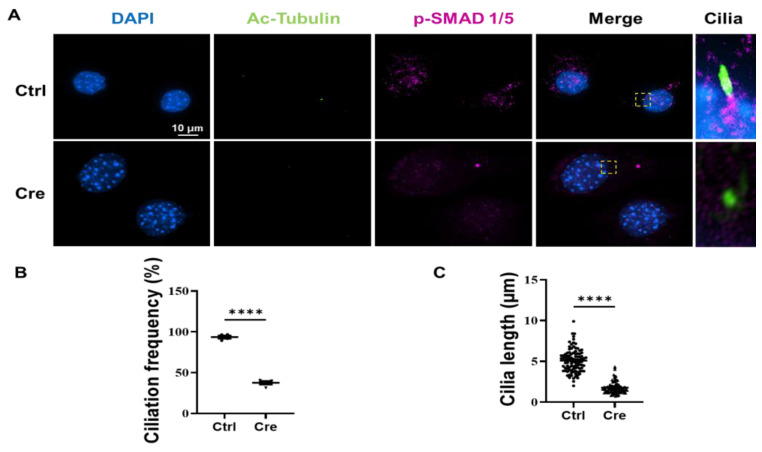
Ciliation frequency and cilia length are decreased in *Scx*-CreERT2;*Ift88*^fl/fl^ tenocytes. Following BMP4 stimulation, isolated tenocytes were fixed and stained for Ac-Tubulin. (**A**) Representative immunofluorescence images showing p-SMAD 1/5 (Magenta) co-localized with Ac-Tubulin (Green) at the ciliary base in control (Ctrl) tenocytes. Nuclei were counterstained with DAPI (Blue). The boxed region indicates cilium, and is shown at higher magnification. (**B**) Quantification of the ciliation frequency revealed a significant decrease in Cre-treated (IFT88 knockdown) tenocytes compared with controls. In total, 100 cells per condition were analyzed for ciliation percentage quantification. (**C**) The ciliary length decreased from 5.09 µm to 1.77 µm with IFT88 knock down. Data is presented as means ± SEM (*n* = 4). Statistical analyses were performed by two-tailed unpaired Student’s *t*-test. **** *p* < 0.0001. In total, 100 cells were measured for cilia length for each condition. Ctrl, Control; Cre, IFT88 knock down. Scale bar: 10 µm.

We stained the isolated tenocytes for Ac-Tubulin (Green), p-SMAD 1/5 (Magenta) and nuclear localization with DAPI (Blue). We found that the p-SMAD 1/5 co-localized with Ac-Tubulin at the ciliary base (Figure 2A). The ciliation frequency of tenocytes decreased from 94% to 38% with IFT88 knock down (Figure 2B). In addition, the ciliary length decreased from 5.09 µm to 1.77 µm (Figure 2C). More cells are shown in the Supplementary Figure (Figure S1).

### 3.3. BMP Signaling Is Inhibited by Dysfunctional Primary Cilia

**Figure 3:**
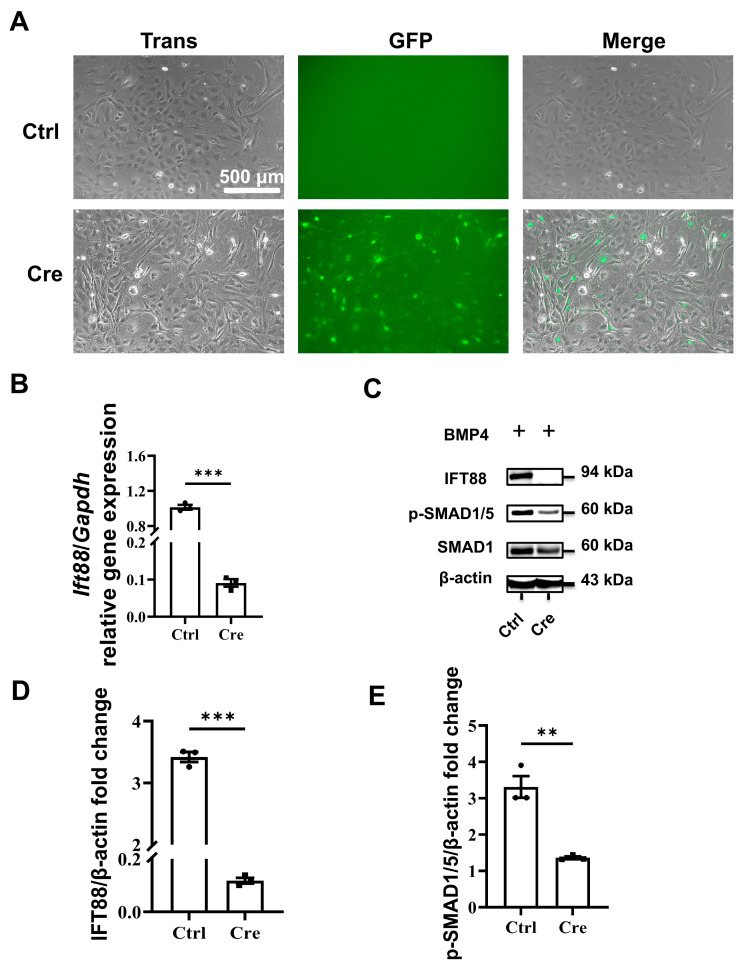
BMP signaling is inhibited in tenocytes with dysfunctional primary cilia. Isolated tenocytes were treated with Cre-GFP adenovirus at an MOI of 1000. (**A**) About 50% of primary tenocytes cells expressed GFP. Cells were uniformly stimulated with BMP4 (50 ng/mL) for 1 h to activate canonical BMP signaling prior to analysis. (**B**) The *Ift88* expression was significantly inhibited in tenocytes transfected with Cre adenovirus. (**C**) IFT88 protein was substantially reduced from the Ade-Cre-virus-treated tenocyte cultures. The Smad 1/5 phosphorylation level decreased significantly in the Cre-treated tenocytes. (**D**) The quantification of IFT88 level normalized to β-actin level (from Western blot in (**C**)). (**E**) The quantification of SMAD 1/5 phosphorylation level normalized to β-actin (from Western blot in (**C**)). Data is presented as means ± SEM (*n* = 3). Statistical analyses were performed by two-tailed unpaired Student’s *t*-test. ** *p* < 0.01; *** *p* < 0.001.

We then treated the isolated tenocytes with Cre-GFP adenovirus at an MOI of 1000. We found that about 50% of primary tenocytes cells expressed GFP (Figure 3A). We also confirmed that the *Ift88* gene expression was significantly inhibited in tenocytes transfected with Cre adenovirus (Figure 3B). By Western blot analysis, we found that the IFT88 protein was reduced in the Cre-treated tenocyte cultures (Figure 3C,D). The p-SMAD 1/5 level decreased significantly in the Cre-treated tenocyte cultures (Figure 3C,E).

### 3.4. Chondrogenic and Osteogenic Gene Expressions Are Inhibited by Dysfunctional Primary Cilia

**Figure 4:**
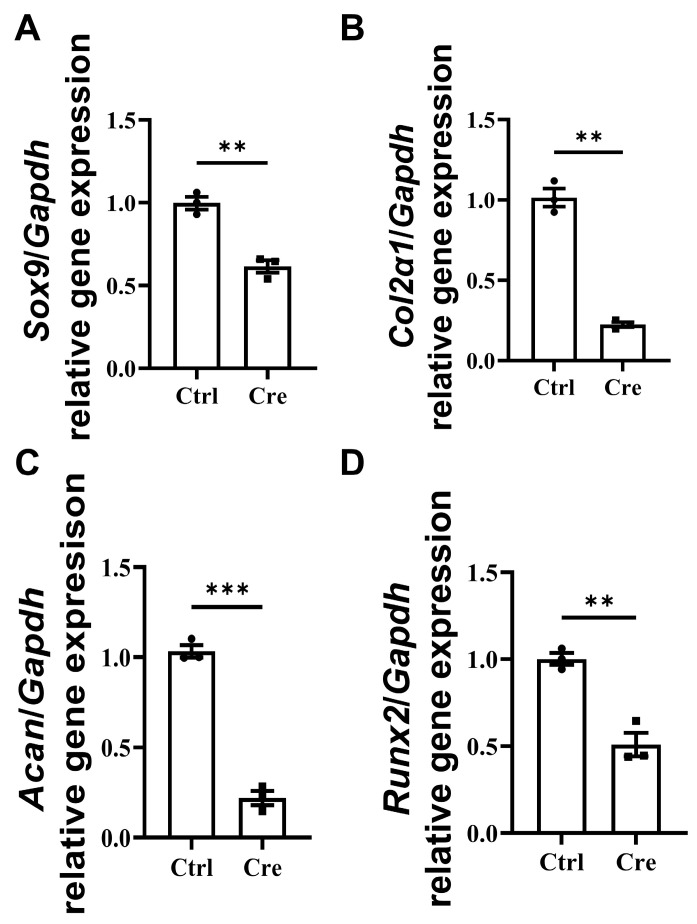
Reduced expression of chondrogenic markers in Cre-treated tenocytes with dysfunctional primary cilia. RT–qPCR analysis of chondrogenic gene expression in control (Ctrl) and Cre-treated tenocytes. Expression levels of *Sox9* (**A**), *Col2α1* (**B**), *Acan* (**C**), and *Runx2* (**D**) were significantly reduced in the Cre-treated tenocytes compared with controls. Gene expression was normalized to *Gapdh* housekeeping gene expression and presented relative to the control group. Data are shown as mean ± SEM (*n* = 3). Statistical analyses were performed by two-tailed unpaired Student’s *t*-test. ** *p* < 0.01, *** *p* < 0.001.

Primary tenocytes were isolated from *Scx*-CreERT2;*Ift88*^fl/fl^ mice and infected with Cre-GFP adenovirus at a multiplicity of infection (MOI) of 1000. Quantitative RT–qPCR analysis demonstrated that deletion of *Ift88* significantly suppressed chondrogenic and osteogenic gene expression. Specifically, the mRNA levels of chondrogenic marker genes, including *Sox9*, *Col2a1*, and *Acan*, were markedly downregulated in Cre-treated tenocytes compared to controls (Figure 4A–C). In addition, the expression of the osteogenic marker gene *Runx2* was also significantly reduced in Cre-treated tenocytes (Figure 4D), suggesting impaired chondrogenic and osteogenic differentiation capacity. Collectively, these findings indicate that *Ift88* deletion disrupts primary cilia function and attenuates both chondrogenic and osteogenic gene expression in primary tenocytes treated with Cre-GFP adenovirus at a multiplicity of infection (MOI) of 1000.

### 3.5. Traumatic HO Is Diminished in Mice with Dysfunctional Primary Cilia

**Figure 5:**
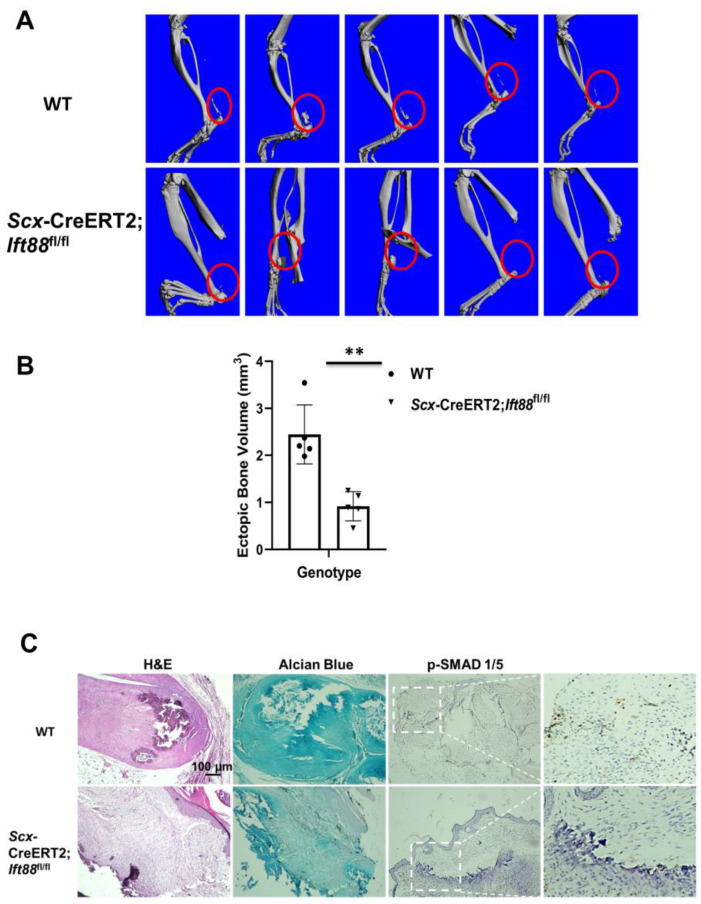
Traumatic HO is diminished in mice with dysfunctional primary cilia. (**A**) Two months after surgery, legs were harvested and examined with µCT. HO sites are marked with red circles. (**B**) The quantification of the HO volume measured by µCT. (**C**) H&E staining showed the confirmed bone structure in the harvested tendons from WT mice or *Scx*-CreERT2;*Ift88*^fl/fl^ mice. Alcian blue staining indicated the cartilage formation in the *Scx*-CreERT2;*Ift88*^fl/fl^ mice compared to WT mice. Phosphorylated SMAD 1/5 immunostaining revealed the p-SMAD 1/5 levels in *Scx*-CreERT2;*Ift88*^fl/fl^ mice compared to WT mice. The boxed region indicates the p-SMAD 1/5, and is shown at higher magnification. Data is presented as means ± SEM (*n* = 5). Statistical analyses were performed by two-tailed unpaired Student’s *t*-test. ** *p* < 0.01. Scale bar: 100 µm.

To examine the effects of dysfunctional cilia on traumatic HO formation, we crossed *Scx*-CreERT2 mice with *Ift88*^fl/fl^ mice to derive homozygous *Scx*-CreERT2;*Ift88*^fl/fl^ mice (Figure 5A). Two months after burn–tenotomy injury, we found that, compared to WT, HO volume decreased in *Scx*-CreERT2;*Ift88*^fl/fl^ mice with dysfunctional cilia (Figure 5B). The H&E staining confirmed normal endochondral bone formation at ectopic sites in tendons harvested from WT mice, whereas tendons from *Scx*-CreERT2;*Ift88*^fl/fl^ mice exhibited reduced bone formation. The Alcian blue staining suggested reduced cartilage-associated staining in the tendon region of Ift88 mutant animals. Phosphorylated SMAD 1/5 immunostaining revealed reduced p-SMAD 1/5 signal intensity in *Scx*-CreERT2;*Ift88*^fl/fl^ mice compared to WT mice (Figure 5C).

## 4. Discussion

Dysfunction of primary cilia causes a broad spectrum of human diseases that are collectively termed ciliopathies. For example, skeletal abnormalities, such as oral–facial malformation, short ribs/limbs, and polydactyly, are commonly associated with ciliopathies [41]. In FOP, a hallmark sign is congenital great toe malformation, but other joint malformations also occur prenatally [2]. In addition, the population and ciliation ratio of fibro/adipogenic progenitors (FAPs), one major cell types initiating HO formation in FOP [14,15], sharply increases upon muscle injury [42,43]. Recently, several studies associated primary cilia with TGF-β signaling [44] and BMP signaling in mammalian cells [45,46,47]. Also, loss of CEP128, a centriole subdistal appendage protein required for regulating ciliary signaling, leads to impaired TGF-β/BMP signaling, especially phosphorylation of Smad 2/3 in zebrafish [48].

**Figure 6:**
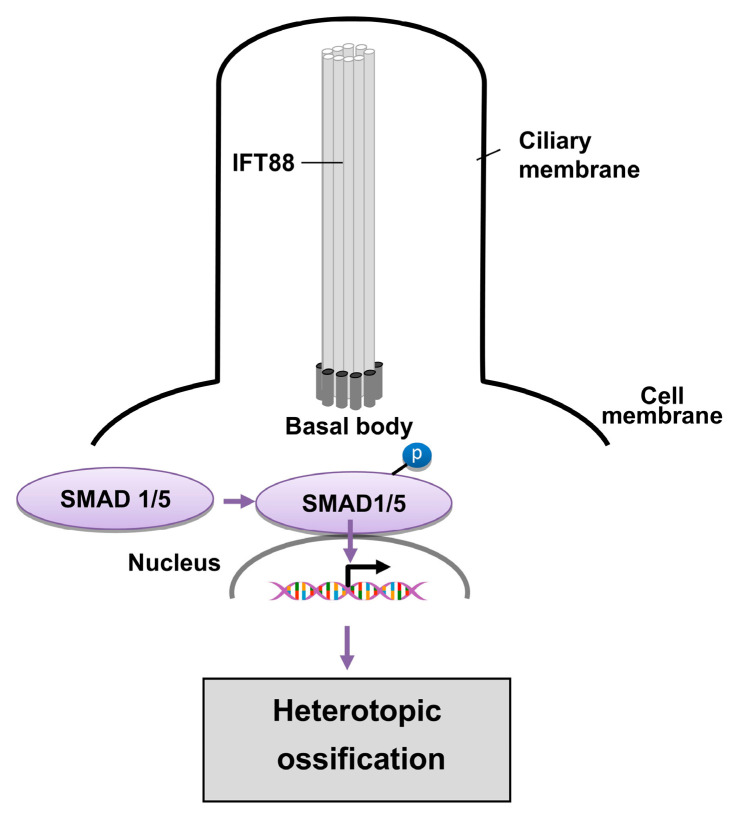
Primary cilia regulate traumatic HO. Schematic illustrating the role of primary cilia in BMP–SMAD signaling during traumatic HO. The ciliary protein IFT88 is required for the formation and function of the primary cilium, which acts as a signaling hub at the cell membrane. Upon activation of BMP signaling, SMAD1/5 becomes phosphorylated (p-SMAD1/5) near the basal body of the cilium. Phosphorylated SMAD1/5 translocates to the nucleus to regulate transcription of osteogenic genes, ultimately promoting ectopic endochondral bone formation and the development of heterotopic ossification.

In this study, we show that there is a strong association between BMP signaling and the integrity of primary cilia. Co-localization of Ac-Tubulin and p-SMAD 1/5 in WT tenocytes and their abrogation with IFT88 knockdown in vitro suggests an important localized structural relationship. Reduced BMP signaling after IFT88 knockdown further suggests a key structure–functional relationship. Furthermore, using homozygous *Scx*-CreERT2;*Ift88*^fl/fl^ mice, we found that traumatic HO can be mitigated in a model of dysfunctional cilia. Taken together, we hypothesize that functional primary cilia play a crucial role in mediating trauma-induced HO through the regulation of BMP signaling (Figure 6).

BMP signaling is involved in the formation of traumatic HO is supported by other evidence. For example, Activin A is expressed in response to injury in both FOP and traumatic HO models, but by different types of cells. Although wild type ACVR1 does not transduce signal when engaged by activin A, activin A is nevertheless expressed in traumatic HO. Anti-activin A does not abrogate traumatic HO, whereas antibodies that neutralize ACVR1 or ALK3-Fc (which blocks osteogenic BMPs) do partly mitigate formation of ectopic bone [49].

The reduction of HO in the *Scx*-CreERT2;*Ift88*^fl/fl^ model is robust, but not complete, suggesting that there are other signaling pathways involved in the process of traumatic HO. Indeed, traumatic HO arises through the aberrant activation of multiple osteogenic and inflammatory signaling pathways following severe tissue injury. The early inflammation activates TGF-β signaling, which is considered one of the primary drivers for osteogenic differentiation of mesenchymal progenitors in ectopic endochondral ossification [50]. Concurrently, hypoxia in the injured soft tissue stabilizes HIF-1α, which enhances chondrogenic commitment and synergizes with BMP signaling to promote endochondral ossification [39,51,52]. Additional developmental pathways—including Wnt/β-catenin, Hedgehog, and mTOR—further regulate progenitor expansion, chondrogenesis, and bone formation [36,52,53]. Traumatic HO is regulated by the PTEN/AKT signaling pathway in osteogenic and chondrogenic differentiation of tendon-derived stem cells [54]. It is plausible that primary cilia contribute to the pathological regulation of tendon-derived stem cells by interacting with PTEN/AKT signaling. Coordination among these inflammatory and osteogenic signaling networks form the molecular basis of HO pathogenesis and represent key targets for therapeutic intervention.

Primary cilia function not only as a mechanosensory hub for BMP signaling, but also as a critical regulator of cell proliferation, migration, and lineage commitment. Through their role in coordinating multiple developmental pathways—including Hedgehog, Wnt, and PDGFRα signaling—primary cilia help control the balance between progenitor cell expansion and differentiation [55]. Disruption or heightened sensitivity of ciliary signaling can therefore alter the activation state of mesenchymal progenitors and chondro-osteogenic programs, creating a permissive environment for endochondral ossification. Given these integrative roles, abnormalities in primary cilia structure or signaling may significantly influence susceptibility or progression of HO.

In summary, our findings indicate that primary cilia dysfunction is associated with reduced traumatic HO, at least partly through impaired BMP signaling. Given their central role in coordinating multiple signaling pathways, changes in primary cilia structure or function may contribute to HO susceptibility or progression and warrant further exploration as potential therapeutic targets.

## Figures and Tables

**Table 1:** Primer sequences used in the RT-qPCR.

Gene Name	Sequence
*Gapdh* F	TGAAGGTCGGTGTGAACGGATTTGGC
*Gapdh* R	CATGTAGGCCATGAGGTCCACCAC
*Scx* F	AGCCCAAACAGATCTGCACCTT
Scx R	CTTCCACCTTCACTAGTGGCATCA
*Tnmd* F	TGTACTGGATCAATCCCACTCT
*Tnmd* R	GCTCATTCTGGTCAATCCCCT
*Myod1* F	CTGCTCTGATGGCATGATGGAT
*Myod1* R	ACTGTAGTAGGCGGTGTCGT
*Myog* F	CAACCAGGAGGAGCGCGATCTCCG
*Myog* R	GCTGGGTGTTAGCCTTATGTGAATGG
*Ift88* F	TCCAACTGATTCCCAAGCTC
*Ift88* R	TGGCACTCAGTCGTTCACTC
*Sox9* F	GCGTGCAGCACAAGAAAGAC
*Sox9* R	TCCGTTCTTCACCGACTTCCT
*Col2α1* F	CGAGTGGAAGAGCGGAGACTA
*Col2α1* R	GAAAACTTTCATGGCGTCCAA
*Acan* F	CCTGCTACTTCATCGACCCC
*Acan* R	AGATGCTGTTGACTCGAACCT
*Runx2* F	CAGATCCCAGGCAGGCACAGTC
*Runx2* R	ACAGCGGCGTGGTGGAGTG

## Data Availability

The raw data supporting the conclusions of this article will be made available by the authors on request.

## Supplementary Materials

The following supporting information can be downloaded at: https://www.mdpi.com/article/10.3390/biomedicines14030712/s1, Figure S1: Representative immunofluorescence images showing p-SMAD1/5 colocalized with Ac-Tubulin at the ciliary base.

## Abbreviations

HO	Heterotopic ossification
FOP	Fibrodysplasia ossificans progressiva
BMP	Bone morphogenetic protein
ACVR1	Activin receptor A type I
TGF-β	Transforming growth factor-beta
IFT	Intraflagellar Transport
FAPs	Fibro/adipogenic progenitors
SMAD 1/5	Mothers against decapentaplegic homolog 1/5
