# Supplementary material for: Primary Cilia Are Required for Efficient BMP Signaling in Traumatic Heterotopic Ossification
Source: Biomedicines. 2026 Mar 19;14(3):712. doi: 10.3390/biomedicines14030712 (PMC13023636; doi:10.3390/biomedicines14030712)

Supplementary Figure.

Representative immunofluorescence images showing p-SMAD1/5 (Magenta) colocalized with Ac-Tubulin (Green) at the ciliary base in control (Ctrl) tenocytes. Nuclei were counter-stained with DAPI (Blue). The boxed region indicates cilium and is shown at higher magnification.

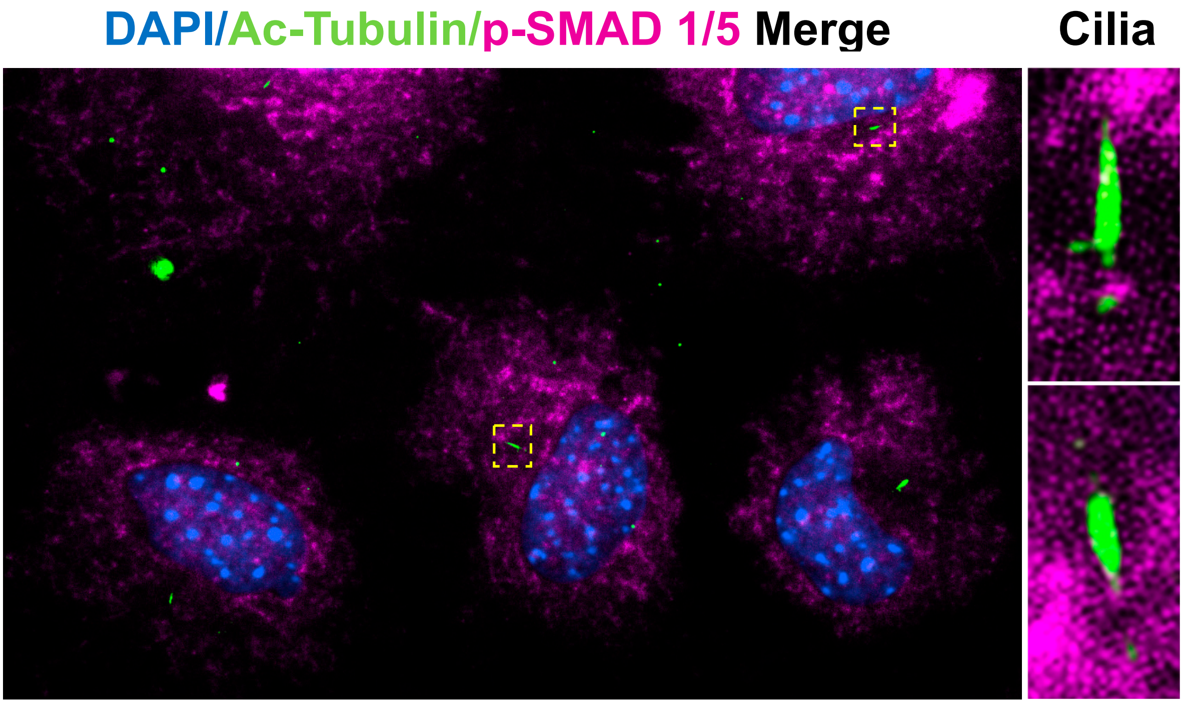

Supplement: Supplementary file 1 [file biomedicines-14-00712-s001.zip › biomedicines-4085751-supplementary.pdf]
